# XGBMUT: Predicting the Functional Impact of Missense Mutations Using an Extreme Gradient Boost Classifier

**DOI:** 10.1021/acsomega.4c10179

**Published:** 2025-02-19

**Authors:** Gabriel Rodrigues Coutinho Pereira, Loiane Mendonça Abrantes Da Conceição, Bárbara de Azevedo Abrahim-Vieira, Carlos Rangel Rodrigues, Lucio Mendes Cabral, Ricardo Limongi França Coelho, Joelma Freire De Mesquita

**Affiliations:** 1 Laboratory of Molecular Modeling and QSAR, Faculty of Pharmacy, Federal University of Rio de Janeiro, 373 Carlos Chagas Filho Avenue, Rio de Janeiro, Rio de Janeiro 21941-170, Brazil; 2 Laboratory of Industrial Pharmaceutical Technology, Faculty of Pharmacy, Federal University of Rio de Janeiro, 373 Carlos Chagas Filho Avenue, Rio de Janeiro, Rio de Janeiro 21941-170, Brazil; 3 Laboratory of Bioinformatics and Computational Biology, Biomedical Institute, Federal University of the State of Rio de Janeiro, 296 Pasteur Avenue, Rio de Janeiro, Rio de Janeiro 22290-240, Brazil; 4 Faculty of Business Administration, Accountability and Economics, Federal University of Goiás, Samambaia Street, Goiânia, Goiás 74001-970, Brazil

## Abstract

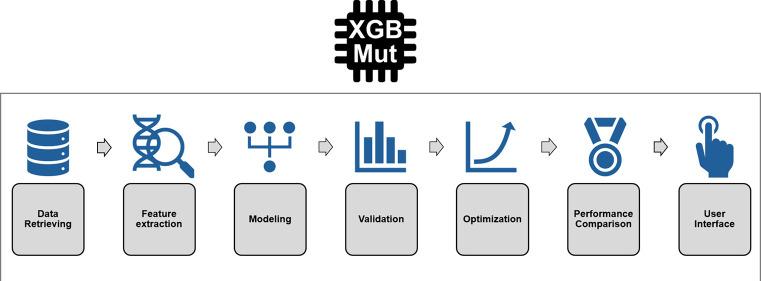

Millions of new mutations have been discovered largely due to advancements in genome projects, but characterizing their effects through traditional wet-lab experiments remains labor-intensive and time-consuming. Functional prediction algorithms offer a solution by enabling the efficient screening of mutations, thereby saving time and resources. The objective of this study was to develop a competitive algorithm for predicting the functional impact of missense mutations. A unified database and substitution matrices containing predictor variables specifically for missense mutations were initially constructed. Subsequently, values for the predictor variables were collected from the training and test sets derived from the ClinVar and HumsaVar databases. A series of supervised machine learning classifiers were then trained, and their performance was evaluated using the test set. The best-performing model was additionally compared against ten currently available functional prediction algorithms. The proposed algorithm, XGBMut, demonstrates exceptional accuracy in classifying missense mutations while also exhibiting a competitive performance. Additionally, a user-friendly graphical interface was developed to enhance accessibility for professionals in various fields. Unlike most existing methods, XGBMut eliminates the need for a web server dependency and the installation of third-party software, making it a more versatile tool for users.

## 1 Introduction

Single nucleotide variants (SNVs) are the most frequent forms of genetic mutations in humans. SNVs occur when a single nucleotide is substituted for another in a given DNA sequence (DNA). These changes can affect coding regions of DNA, which contain the genetic information necessary for protein production, or noncoding regions. Coding region SNVs can be further subdivided into synonymous and nonsynonymous variants. Nonsynonymous SNVs, also known as missense mutations, are responsible for altering the amino acid sequence of proteins,^1^ which can ultimately affect protein structure and function, leading to the development of diseases.^2,3^ A well-known example is sickle cell anemia, a form of hemoglobinopathy caused by a missense mutation in the HBB gene. This mutation causes a substitution of glutamic acid with valine at position 6 (E6 V), resulting in the production of abnormal hemoglobin (HbS). The presence of HbS causes red blood cells to adopt a sickle shape, impairing oxygen transport and leading to a range of clinical complications.^4^ Thanks to next-generation sequencing technologies, millions of new SNVs have been discovered, which have enabled the development of human genome projects, especially since the 2000s. However, understanding the impact of missense mutations using laboratory protocols is laborious and time-consuming, particularly given the vast amount of available genetic data.^5^ Thus, determining the functional effects of genetic variants remains a significant challenge in human genetics. Among the more than 4 million missense variants identified to date, only roughly 2% have been clinically categorized as pathogenic or benign, with the majority remaining classified as variants of uncertain significance. This uncertainty poses obstacles to diagnosing rare diseases and developing targeted treatments for genetic conditions.^6^

In this scenario, computational methods allow for a faster and more efficient prediction of mutation effects, aiding in the prioritization of the most likely pathological mutations for laboratory analysis. Functional prediction is an effective, widely used, and indispensable approach for characterizing the effects of missense mutations, advancing studies of various human genetic and metabolic disorders. Among the currently available algorithms, SIFT, PolyPhen2, and MutationTaster are widely used for mutation screening, guiding clinical and laboratory studies.^5^ Essentially, functional prediction algorithms are supervised machine learning (ML) models designed to classify new observations by extracting patterns from databases of mutations already classified through laboratory experiments. These algorithms determine whether a given mutation is neutral or deleterious (pathogenic) based on the characteristics of the affected protein and the specific amino acid substitution.^7,8^

Well-established functional prediction methods, which were developed during the 2000s and 2010s typically rely on traditional ML classifiers, such as Naïve Bayes (e.g., PolyPhen2), Hidden Markov Models (e.g., Panther, FATHHM), random forests (e.g., MutPred, nsSNPAnalyzer), support vector machines (e.g., SNPs&GO, PhD-SNP), and shallow neural networks (e.g., SNAP).^9^ With the rise of deep learning over the past decade and the more recent advancements in language models, new functional prediction algorithms, including MutPred2,^10^ VariPred,^11^ MutFormer,^12^ MetaRNN,^13^ and AlphaMissense^6^ have been developed based on these state-of-art approaches. Methods such as VariPred and MutFormer use protein data representation techniques inspired by natural language processing (NLP). Specifically, the transformer neural network architecture is employed to learn context-sensitive representations from amino acid sequences. Despite the use of more advanced algorithms, there has been only a modest improvement in their overall performance compared to traditional classifiers, with the trade-off being a substantial increase in computational demand.^10−12^ In addition to the aforementioned techniques, ensemble methods have also been proposed for predicting the functional effects of missense variants. These models, which include REVEL (The Rare Exome Variant Ensemble Learner)^14^ and PredictSNP,^15^ integrate scores from multiple pathogenicity prediction tools. For instance, REVEL combines 18 individual pathogenicity scores derived from 13 different tools, while PredictSNP combines six functional prediction methods into a consensus classifier.

Key features considered by these algorithms encompass the potential functional impacts of variants, which can result in a range of molecular alterations. These may include potential disruptions to protein structure, such as alterations in secondary or tertiary structure, changes in stability, interference with macromolecular interactions (e.g., metal-binding or nucleic acid binding), loss of posttranslational modifications (e.g., SUMOylation, acetylation), dysfunction in catalytic or allosteric mechanisms, and impacts on the intrinsic disorder or protein folding.^10^ Additionally, nearly all traditional functional classifiers incorporate amino acid substitution frequencies, residue similarity, and evolutionary conservation—a widely recognized indicator of functional importance—as key factors in their predictions.^11,16^

Many current approaches, despite incorporating both local and global descriptors related to protein function, often fail to offer detailed insights into the underlying mechanisms affected by mutations. These methods tend to operate as black boxes, not generating actionable hypotheses regarding the molecular consequences of the variants, thereby limiting their ability to drive further biological understanding.^10^ Thus, it is common practice to complement functional predictions with parallel assessments using software tools that predict the impact of related protein features,^17,18^ such as protein stability (e.g., I Mutant 3.0,^19^ FoldX^20^), post-translational modifications (e.g., SUMOylation: Deep-Sumo,^21^ acetylation^22^), and phenotypic traits like aggregation propensity, amyloid formation, and chaperone binding.^23^ This multifaceted approach helps to provide a more comprehensive understanding of the potential mechanism involved in impaired protein function.

In addition to the heterogeneity of ML algorithms and feature selection strategies employed by functional prediction algorithms, there is considerable variation in the data sets used to construct these models. These data sets, composed of mutations with known effects, are typically derived from major human mutation databases such as ClinVar, Humsavar, 1000 Genomes, COSMIC, SwissVar, or dbSNP. Each of these databases varies in scope and focus, which can influence the predictions made by the models. For instance, COSMIC is dedicated to cancer-related somatic mutations, while 1000 Genomes primarily focuses on population-specific genetic variation.^24^ The variability in the underlying functional prediction methods often results in inconsistent or even contradictory predictions across different methods. Consequently, a common strategy for validating a novel approach is to benchmark its performance against well-established models using large, unseen data sets.^10−12^ Additionally, these major databases are commonly used to benchmark the performance of existing functional prediction classifiers within more specific scenarios such as the evaluation of cancer-related missense mutations. This benchmarking process aims to identify a reference classifier tailored to a specific context, ensuring more accurate and context-relevant predictions.^9,16^

Despite recent advances in ML, which have led to the development of robust models capable of explaining even nonlinear phenomena, none of the currently available methods fully address the complexity and diversity of human genomes.^25^ As a result, no gold-standard method currently exists for predicting the impact of missense mutations, and the performance of these methods can vary significantly depending on the specific context, such as the protein, structural domain, or functional region, in which they are applied.^26^ Additionally, many of these methods lack frequent updates, are hosted on servers with minimal/no user support, or are provided as command-line interfaces with limited documentation and outdated dependencies, making them difficult for nonspecialists to use effectively.

To address these limitations, we propose a novel functional prediction algorithm that offers an integrated, efficient, and open-box approach. Our method, based on an extreme gradient boosting classifier, stands out by combining multiple data sources to provide a comprehensive framework for predicting the effects of missense mutations. Additionally, it provides a detailed output file that includes all predictor variables used in the model’s predictions, enhancing the interpretability of results. XGBMut also features a user-friendly interface that requires no installation of dependencies, facilitating its application by nonspecialists.

Therefore, the objective of this study is to develop, validate, and optimize a competitive functional prediction algorithm for classifying new missense mutations as well as to create software that enables the use of the algorithm through a user-friendly interface. This approach could assist in the initial screening of mutations most likely deleterious, allowing for in-depth study through clinical and laboratory assays,^27^ thereby optimizing time and resources.

## 2 Materials and Methods

### 2.1 Construction of a Unified Database and Substitution Matrices

To characterize human proteins and their amino acid positions affected by mutations, molecular descriptors were obtained by merging the following databases: DescribeProt,^28^ UniProt,^29^ Gene Ontology,^30^ PhosphoSitePlus,^31^ Catalytic Site Atlas,^32^ and Conserved Domain Database.^33^ The integration of these databases, as well as data cleaning and wrangling, were conducted using the Pandas library, in Python.^34^ The protein identification code (UniProt ID) column was used for record matching. A detailed description of the data sets used and the information retrieved from them is provided in File S1.

Additional features related to amino acid substitutions were derived by utilizing a series of 21 substitution matrices, each of size 20 × 20, corresponding to the total number of possible amino acid substitutions. The features selected for constructing the confusion matrices were defined based on the physicochemical properties and probability distributions of amino acid substitutions, which may potentially contribute to the deleterious impact of missense mutations.^28,35^ A detailed description of the constructed substitution matrices, along with specific information extracted from them, is available in File S2.

### 2.2 Preparation of Training and Validation Sets

Experimentally classified mutations were obtained from the ClinVar^36^ and HumsaVar^24^ databases, selected as the training and validation sets, respectively. A filter was initially applied to select only missense mutations in the ClinVar database, which contains various classes of genetic mutations. Additionally, mutations with uncertain or conflicting clinical significance were removed, resulting in 180,680 mutations. Mutations characterized as pathogenic or possibly pathogenic were labeled as deleterious, while those classified as benign or possibly benign were labeled as neutral.

The HumsaVar database contains 71,210 missense mutations already classified as pathogenic, possibly pathogenic, benign, or possibly benign. The same criteria used for ClinVar were applied to label HumsaVar variants as neutral (benign or possibly benign) or deleterious (pathogenic or possibly pathogenic).

Unnecessary and redundant columns were removed from the databases, resulting in three columns: the class label, the mutation in single-letter amino acid format, *e.g.,* A4 V, where the first letter is the native amino acid, followed by the affected position and the mutated amino acid), and the UniProt ID of the affected protein. Rows with missing values were eliminated, and redundant mutations across both databases were removed. To balance the training set, the minority class was oversampled using the *over_sampling* function from the *imblearn* library, which involved randomly drawing samples from the less frequent class with replacement (bootstrap).^37^

### 2.3 Automated Extraction of Predictor Variables

To obtain values for the 61 predictor variables for each mutation in the training and test sets, we developed a function using native Python functions and the Pandas library.^34^ The function begins by iterating through a data set of mutations to identify the affected protein using its UniProt ID and the specific mutation position. The function initially checks whether the provided native amino acid matches the protein sequence and validates the mutation format, discarding any invalid mutations. Once validated, the function searches through predictor variables in the unified database for the corresponding values at the mutation position. It then utilizes the native and mutated amino acids to extract values for twenty-two predictor variables from 20 × 20 substitution matrices, retrieving the value based on the native amino acid row and mutated amino acid column.

### 2.4 Predictive Modeling and Validation

The initial step in developing the predictive model for classifying new mutations utilized an automated machine learning (autoML) approach with the PyCaret library in Python. This involved evaluating 15 different model types to identify the best-performing algorithm.^38^ As previously described, the training set was derived from ClinVar, and the test set was from HumsaVar. Following this, an initial round of training and testing was conducted.

Then, to address scaling challenges inherent to methods within the PyCaret pipeline, such as support vector machines and k-nearest neighbors, min–max scaling was applied to the quantitative variables using Scikit-learn, normalizing values between zero and one.^39^ The scaled data sets were then subjected to another round of autoML in Pycaret, following the same methodology previously described.

The scaled data set served as the foundation for training and validating neural networks developed using the TensorFlow and Keras libraries.^39^ An object of the “Sequential” class was initially created. Dense input, hidden, and output layers were added to this object using the “add” function. Then, the “compile” function was used to combine the layers, and the “fit” function was used to train the model. ReLU activation functions were selected for the hidden layers, while the sigmoid function was selected for the output layer. A learning rate of 0.003 and 200 training epochs were used. Five different architectures were tested: (6, 6), (12, 8), (8, 8, 8), (8, 6, 4), and (12, 8, 6, 4). Validation and test set accuracy were monitored during the process.

Finally, the generated models were evaluated on the validation set using performance metrics for the binary classification task: accuracy, area under the ROC curve (AUC-ROC), recall, precision, and F1 score, which were calculated with the corresponding function from the Scikit-learn package. The model with the highest accuracy among those generated was selected for a grid search with 4-fold cross-validation to find the hyperparameters that best optimize the model performance.

Since the model with the best predictive performance was attributed to the extreme gradient boosting class, the grid search was conducted using the GridSearchCV function from the Scikit-learn library^40^ and the “XGBClassifier” function from the XGBoost library.^41^ The grid search encompassed the following hyperparameters: learning rates of 0.01, 0.1, and 0.3; maximum tree depths of 5, 7, and 10; L2 regularization values (lambda) of 0.1, 1, and 10; a range of estimators set at 100, 200, and 500; and percentages of predictor variables used in each tree of 0.5, 0.7, and 1. The selection of the optimal model during the grid search was based on accuracy as the primary criterion.

After identifying the model with the optimal hyperparameter combination, predictor variable selection was conducted using the recursive feature elimination (RFE) method.^42^ This process employed the RFE function from the Scikit-learn library.^40^ A series of configurations for the predictor variables was assessed, ranging from 5 to 55 in increments of 5, with three variables systematically removed at each iteration of the method.

The performance of each model generated from RFE was evaluated on the test set, assessing accuracy, AUC-ROC, recall, precision, and F1-score metrics using functions from the Scikit-learn package, as previously described. Additionally, a confusion matrix was generated to compute the percentages of true negatives, true positives, false positives, and false negatives, utilizing functions from the Scikit-learn library.^40^ The final model was selected based on the combination of the aforementioned metrics. The significance of the predictor variables in the final model, henceforth referred to as XGBMut, was assessed using the “plot_importance” function from the XGBoost library.^41^

### 2.5 Comparative Performance Analysis of XBGMut against Functional Predictive Algorithms Currently Available

**Figure 1:**
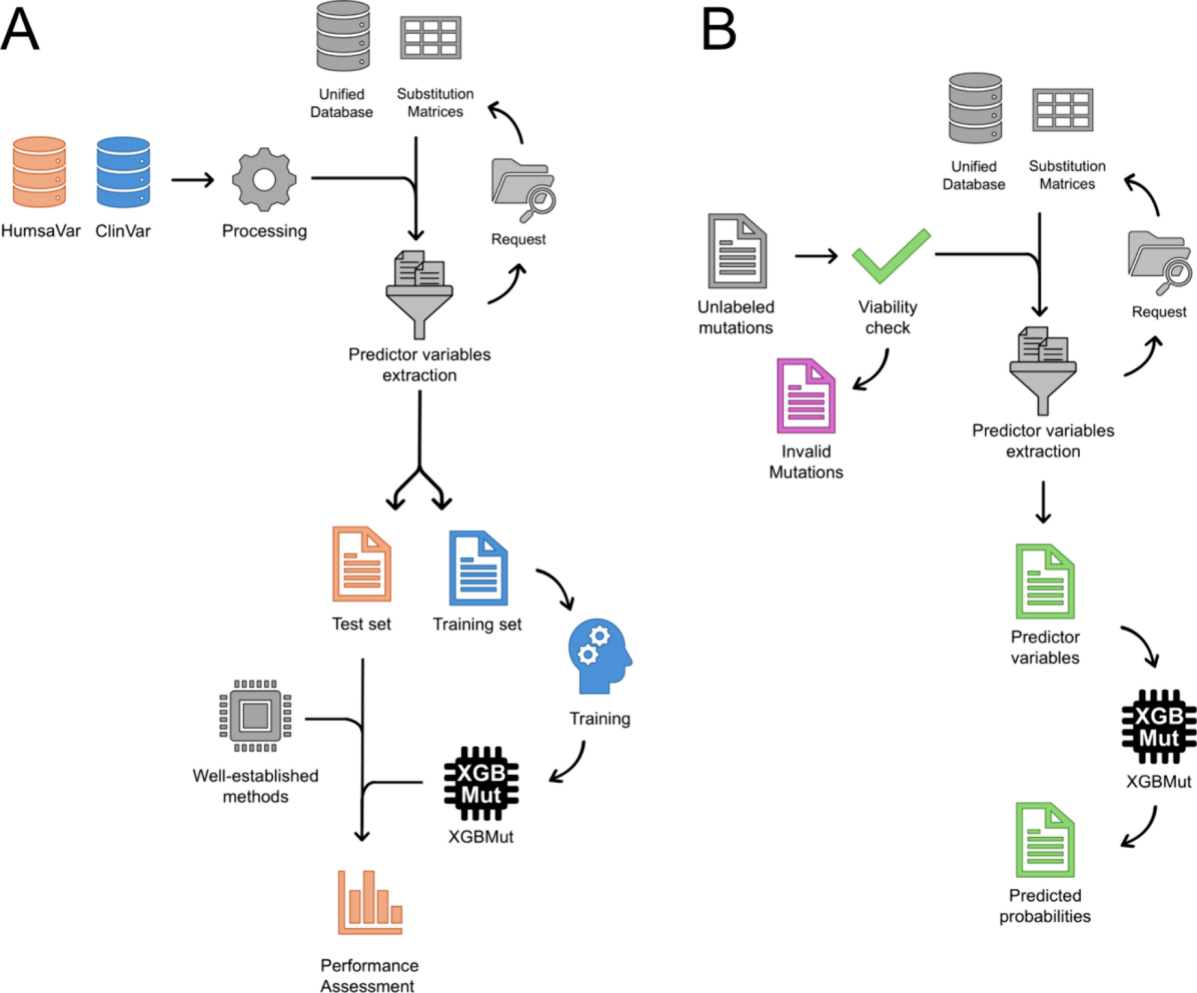
Workflow for data processing, model training, validation, and functioning of the XGBMut classifier. (A) Workflow for XGBMut construction, validation, and benchmarking. The ClinVar and HumsaVar databases were initially processed to remove duplicates and select valid missense mutations for constructing the training and test sets, respectively. These cleaned data sets were then submitted to a function designed to automatically extract local and global predictor variables for the corresponding mutations, based on information from the previously prepared unified database and substitution matrices. The training set was used to develop the XGBMut model, while the test set, containing unseen mutations, was independently evaluated to assess the model’s performance. Finally, the performance of well-established methods for predicting the functional effects of missense mutations was compared to that of XGBMut using the test set, ensuring the model’s viability. (B) Workflow for XGBMut functioning. For unlabeled mutations, an initial viability check ensures that only valid mutations proceed to predict the variable extraction. Invalid mutations are filtered out during this step and saved in a separate file for user reference. Valid mutations were then submitted to a function designed to automatically extract local and global predictor variables for the corresponding mutations based on information from the previously prepared unified database and substitution matrices. Once predictor variables are fully extracted from the submitted data, they are input into the XGBMut model, which predicts the deleteriousness probabilities for each mutation. These predictions are then saved to an output file. Arrows represent the data flow, while icons illustrate the computational processing and model implementation steps.

The performance of the XGBMut model on the test set obtained from the HumsaVar database was compared with that of ten widely used software tools: PANTHER,^43^ SNPs&GO,^44^ PolyPhen-2,^45^ SIFT,^46^ PROVEAN,^47^ FATHMM,^48^ Pmut,^8^ PON-P2,^49^ VariPred,^11^ and MutPred2.^7^ A flowchart detailing all of the steps involved in the training and testing of the proposed model, along with the comparison process with currently available software for predicting the impact of missense mutations, is presented in Figure 1A.

The methods PROVEAN, SIFT, PolyPhen-2, PMut, PON-P2, and FATHMM were used through batch analysis on their respective online servers by selecting the default settings. In contrast, MutPred2.0 was executed in-house, following the recommended parameters outlined in the algorithm documentation.^7^ SNPs&GO and PANTHER were used via a Docker container.^44^ Finally, the in-house VariPred software, available in its corresponding GitHub repository, was used for batch analysis with the default settings selected. Notably, VariPred employs a state-of-the-art, language-based model, making it a robust tool for validating the viability of our proposed software, XGBMut.^11^ The same validation metrics previously described for the XGBMut model were computed for each of the 10 algorithms tested. Additionally, prediction coverage—defined as the percentage of total mutations classified—was computed.

### 2.6 Optimization of the Algorithm and Development of the XGBMut Software

To optimize the execution time and Random Access Memory (RAM) usage, a series of adjustments were made to the functions and databases used by the algorithm. Initially, all predictive variables not included in the final model (post-RFE) were removed from the unified database. The resulting database was then divided into smaller *.json files based on the type of variable or nature of the stored information. The automated predictor variable extraction function was then adapted to contain only the variables selected in the RFE stage. The function was also refactored. The final model was saved in a *.json file, allowing it to be loaded whenever necessary.

The Time library in Python was used to compare the execution time of the original algorithm version with its optimized version in analyzing the test set derived from the HumsaVar database. The operational procedures of the algorithm are illustrated in Figure 1B.

To facilitate the distribution and utilization of the algorithm by third parties, two alternative user interfaces were developed: a graphical user interface (GUI) and a command-line interface (CLI). The graphical interface was developed using the PySimpleGUI library (https://www.pysimplegui.org/), while the command line interface was developed using the Click library in Python (https://click.palletsprojects.com/). The PyInstaller package was used to generate executable files compatible with Windows and Linux (Ubuntu) operating systems. PyInstaller compiles Python applications and all their dependencies into a single package, allowing users to run the application without needing to install a Python interpreter or any modules (https://pyinstaller.org/).

## 3 Results and Discussion

### 3.1 Construction of the Unified Database and Substitution Matrices

The unified database was constructed in a wide format, with observational values organized in rows corresponding to 23,924 different human proteins. The columns encompass the values of 40 predictor variables, the amino acid sequence, and the corresponding gene identifier. A condensed representation of the unified database is provided in Table S1 to exemplify the structure and organization of this data.

A total of 21 substitution matrices (20 × 20) were constructed to store values for an equal number of predictor variables. These matrices were constructed in tab-separated text documents with native amino acids (observations) arranged in rows and mutated amino acids in columns containing the predictor variable values. A reduced version of one of the generated substitution matrices is shown in Table S2 to exemplify their structure and organization.

### 3.2 Obtaining the Training and Test Sets

After the data cleaning process, the test set comprised 69,887 mutations, with 39,263 being neutral and 30,624 deleterious. The training set comprised 72,471 mutations, with 43,168 neutral and 29,303 deleterious mutations. The less frequent class of mutations in the training set, *i.e*., deleterious, was oversampled to balance the number of observations in each class, resulting in a final training set with 86,336 mutations. A reduced version of the training and validation sets is shown in Table S3 to exemplify their structure and organization.

### 3.3 Automated Extraction of Predictor Variables

After processing the training and test sets with the automated function, the columns related to the protein accession code (*ACC*), amino acid substitution (*AA_change*), and experimentally determined class (*Class*) – whether a mutation is neutral or deleterious–were retained from the input file. Additionally, 61 new columns representing predictor variables for each mutation were added to the data set, resulting in a total of 64 columns. A simplified version of the function’s output for the validation set is provided in Table S4 for better understanding.

Variables related to the occurrence of mutations in important protein regions, as well as those reflecting changes in the amino acid’s physicochemical properties, were encoded as binary (dummy) variables, where zero represents the absence of the feature and one represents the occurrence of a specific event. The remaining columns contain integer or floating values corresponding to the predictor variables.

### 3.4 Predictive Modeling and Validation

The data preprocessing stage is detailed throughout the previous sections, concluding with the definition of the training and validation. This section addresses the stages following preprocessing, specifically, the training and validation of machine learning algorithms.

A total of 30 models were generated using the autoML approach, which were ranked according to their accuracy on the validation set. Accuracy is a valid performance metric for classification problems where the data sets are approximately balanced, as in the training set. The performance of the generated models was also analyzed based on the statistical metrics precision, recall, F1-score, ROC-AUC, and Matthews correlation coefficient (MCC), as defined by the following equations:^22,50,51^
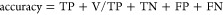
1
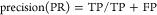
2
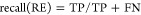
3
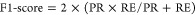
4
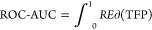
5
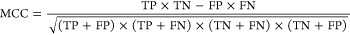
6where TP is the number of true positives, TN is the number of true negatives, FP is the number of false positives, FN is the number of false negatives, PR is precision, RE is recall, and TFP is the false positive rate TPF = (FP/FP + TP).

Accuracy measures the proportion of correct predictions from all predictions made. Precision measures the probability of a correct detection, given that the value is positive, acting as a confidence measure for positive class predictions. Recall, also known as sensitivity or true positive rate, quantifies the proportion of correctly identified positive values out of all actual positives, indicating how many positive observations were missed. The F1-score is a harmonic mean of precision and recall, balancing the two metrics to account for both false positives and false negatives. For these metrics, values closer to 1 indicate better model performance.^52^ ROC-AUC is the area under the curve plotted for recall values against the false positive rate TPF = (FP/FP + TP). ROC-AUC measures the model’s overall ability to discriminate between the two classes at different cutoff points, where a value of 0.5 indicates random discrimination and a value of 1 indicates perfect discrimination between classes.^53^ Finally, MCC was evaluated to provide a robust assessment of the model’s predictive ability across both positive and negative classes.^22,50^ The MCC ranges from −1, indicating complete disagreement between predictions and actual outcomes, to +1, representing a perfect concordance. Values exceeding 0.5 indicate a satisfactory correlation between predictions and actual classes.^54^

Overall, the top 10 best-performing classifiers were ensemble models (Table S5), a class of machine learning algorithms that combine the predictions of multiple weak learners—typically simple, low-performance decision trees—into a stronger model with high predictive power. Among them, only the extra trees model used bagging, where decision trees are trained in parallel with random variations. The remaining algorithms employed boosting, a technique in which decision trees are trained sequentially, with each tree focusing on correcting the errors of its predecessor, thereby progressively reducing residuals and improving performance.^55^

Since PyCaret’s autoML^38^ does not include neural network models—a class known for effectively constructing functional prediction algorithms like SNAP2^56^—five different neural network architectures were explored, and their performance on the test set was assessed. The artificial neural networks exhibited comparable performance across the various architectures with only minor differences noted, as displayed in Table S6. Despite that, their overall performance was lower than those of the bagging and boosting models produced by autoML (Table S5). This suggests that while neural networks are powerful tools, the ensemble methods implemented in Pycaret may offer superior predictive capabilities for the given data set.

Thus, among all of the models tested, the extreme gradient boosting (XGB) model demonstrated the best performance, even without min–max normalization. Extreme gradient boosting (XGB), the method that achieved the best performance (Table S5), is a variation of gradient boosting that has its unique way of building decision trees.^57^ XGB applies regularization and pruning to optimize tree node splits, proving efficient in avoiding overfitting.^58^ As a result, XGB emerged as a robust choice for predictive modeling, demonstrating superior accuracy and reliability in our test case (Table S5).

Given that the XGBoost model was identified as the best-performing class, we conducted an in-house investigation into various hyperparameter combinations for XGBoost (previously described), independent of autoML. Through a grid search analysis with different hyperparameter configurations, we determined the optimal parameter settings to be as follows: learning rate = 0.1, regularization lambda = 1, maximum tree depth = 10, columns included per tree = 0.5, and number of trees = 500. The remaining hyperparameters were set to the algorithm’s default values. The implementation of the model was carried out directly using Python functions from the XGBoost library, thereby ensuring a methodical approach to our predictive modeling efforts.

Building upon this model, we employed the RFE method to eliminate predictor variables that did not significantly contribute to the classification model, which may be considered noise, thus helping to avoid multicollinearity. This approach not only has the potential to enhance model performance but also to reduce its complexity and facilitate interpretation.^59^ Accordingly, we recursively removed the least important predictor variables from the model, ultimately testing 11 simplified model configurations that varied by five in the number of predictor variables included.

The performance of the generated models remains relatively close until the number of predictor variables reaches 15 (Table S7). Below this threshold, there is a notable decrease in the analyzed performance metrics. The model demonstrating the highest values for accuracy, AUC-ROC, recall, precision, and F1-score—thereby demonstrating the best overall performance—used 25 predictor variables. Given the principle of Occam’s Razor, which advocates for simpler models that deliver equivalent results, the model with 25 variables is favored over the more complex full model. Therefore, this model has been designated as the final model for this study and is henceforth referred to as XGBMut.

As shown in Table S7, XGBMut correctly predicted 82.8% of the 69,887 observations in the validation set, which were previously unavailable to the algorithm, demonstrating its high generalization ability for new observations. The model also achieved an AUC-ROC value close to 1 and far from 0.5, indicating its efficiency in distinguishing deleterious from neutral observations. Among all positive predictions made, 82.8% were true positives, as reflected by the calculated precision. Coupled with the recall metric, which indicates that 82.8% of all true positive values were correctly identified, we can conclude that the model demonstrated high confidence in predicting the deleterious class. The calculated F1-score of 82.8% supports this conclusion (Table S7). This is particularly important for functional prediction, where the primary objective is to identify mutations with a higher likelihood of being deleterious for a thorough investigation. Thus, ensuring the reliability of positive class predictions is crucial to avoid unnecessary expenditures of time and resources.^60^

**Figure 2:**
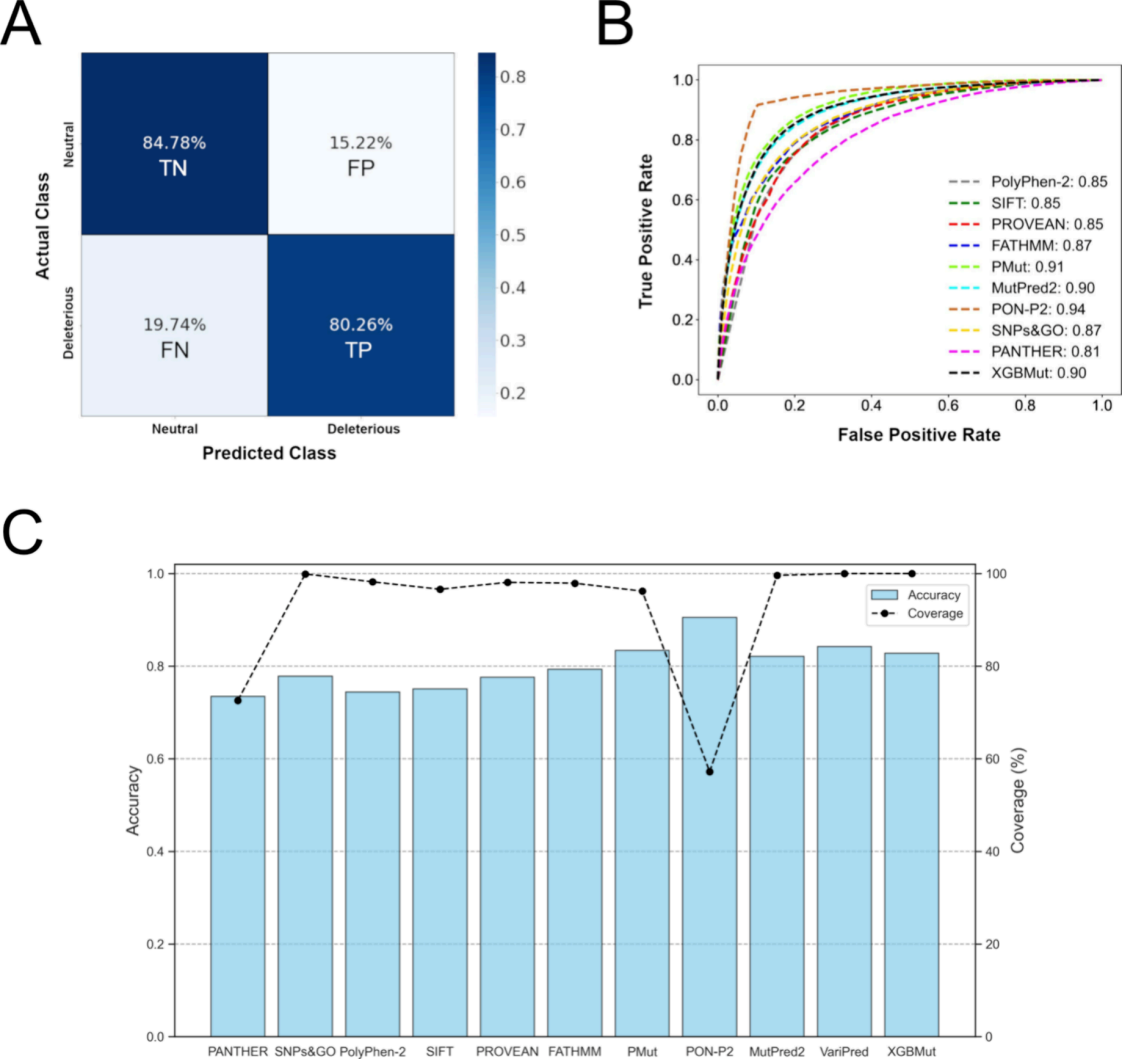
Performance of XGBMut on the test set and its comparison with currently available functional prediction algorithms. (A) Confusion matrix calculated for the final model (XGBMut). TN: true negatives; TP: true positives; FN: false negatives; FP: false positives. (B) ROC curve calculated for XGBMut and ten functional prediction algorithms within the test set. The output of VariPred is not shown, as it is provided as a class label rather than a probability score, making the AUC-ROC calculation infeasible. (C) Blue bars indicate the accuracy of each algorithm, while the dashed black lines represent their corresponding coverage within the test set, i.e., the percentage of total observations (mutations) effectively analyzed by each algorithm.

Furthermore, the MCC computed for the model was 0.651, indicating a strong positive correlation between the predictions and actual classes. This outcome demonstrates the model’s ability to achieve a satisfactory balance in identifying both deleterious and neutral variants, underscoring its overall robustness in classifying the functional effects of missense mutations.^54^ Finally, a confusion matrix for the predictions was provided in Figure 2A to facilitate a clearer understanding of the algorithm’s performance. This is a convenient way to represent the results of a classifier, as all statistical metrics used to evaluate its performance are displayed within it.^61^ The final model correctly classified 84.7% (TN) and 80.2% (TP) of all neutral and deleterious mutations in the validation set, respectively. Nonetheless, the model failed to identify 19.7% of deleterious mutations, misclassifying them as neutral. Only 15.2% of neutral mutations were not identified by the algorithm, which incorrectly classified them as deleterious.

This performance is particularly noteworthy in light of findings by Richards et al., who highlight a significant limitation of most functional prediction algorithms: their low capacity to accurately detect neutral mutations, which can lead to a high number of false positives and misallocation of research resources.^5^ In contrast, the proposed model, XGBMut, demonstrated a commendable ability to avoid this unfavorable characteristic, underscoring its potential as a reliable tool for functional prediction.

We also investigate the contribution of each one of the 25 predictive variables retained in the final model, measured using the F-score, an intrinsic metric of the XGBoost algorithm. The F-score is calculated based on the total number of times a given variable is used as a criterion for class separation in the decision tree nodes of the model.^41^ Analyzing the F-scores provides insights into the variables that are most influential in predicting outcomes, thereby enhancing the understanding of the model’s decision-making process. According to the F-score values (Figure S1), the biological features that most contributed to distinguishing neutral from deleterious mutations were:

(i) *SCRIBER-score*: this score reflects mutation occurrences in amino acids that interact with other proteins.
(ii) *VSL2-score*: this score indicates mutation occurrences in intrinsically disordered regions of proteins.
(iii) Evolutionary Conservation: this is assessed through various metrics, including *Average Conservation, MMSeq2 Conservation Score, MMSeq2 Conservation Level*, and *Conserved Domain*.
(iv) Biological Processes: the protein’s participation in biological processes was expressed by metrics such as *GO Enable* and *GO Involved*.
(v) Physicochemical Alterations: this includes alterations in the amino acid’s physicochemical properties due to mutations, encompassing *hydropathy*, *molecular mass*, and *volume*.
(vi) Likelihood of Substitution: this likelihood is determined by scores such as *PSSM Score*, *BLOSUM Score*, and *Neutral Frequency*.

### 3.5 Comparative Performance Analysis of XBGMut against Functional Predictive Algorithms Currently Available

The performance of the final model, XGBMut, was compared against 10 well-established functional prediction algorithms, including the state-of-the-art, language-based model VariPred, using the same test set derived from the HumsaVar database. This outcome is summarized in Table 1. Conducting such a comparative analysis with other state-of-the-art functional prediction algorithms ensures that the model is assessed within the context of established, state-of-art methods, offering valuable insights into its relative strengths and weaknesses.^7,8^

**Table 1:** Comparison of the Performance of XGBMut with Other Functional Prediction Methods on the Validation Set Derived from HumsaVar

method	coverage	accuracy	precision	recall	F1-score	AUC-ROC	MCC
PANTHER	72.6%	0.735	0.735	0.735	0.735	0.809	0.470
SNPs&GO	99.9%	0.778	0.788	0.778	0.772	0.869	0.541
PolyPhen-2	98.2%	0.744	0.785	0.744	0.742	0.847	0.541
SIFT	96.6%	0.751	0.776	0.751	0.751	0.847	0.530
PROVEAN	98.1%	0.776	0.787	0.776	0.777	0.850	0.562
FATHMM	97.9%	0.793	0.792	0.793	0.793	0.873	0.572
PMut	96.2%	0.834	0.834	0.834	0.834	0.911	0.661
PON-P2	57.2%	0.905	0.906	0.905	0.905	0.939	0.820
MutPred2	99.6%	0.821	0.824	0.821	0.821	0.895	0.650
VariPred	100%	0.842	0.827	0.805	0.816		0.678
XGBMut	100%	0.828	0.828	0.828	0.828	0.898	0.651

Through an extensive analysis using a validation set of approximately 70,000 mutations, the XGBMut algorithm exhibited overall performance that was comparable to, and in several cases surpassed, many of the leading open-access functional prediction algorithms for missense mutation screening. As detailed in Table 1, XGBMut outperformed several well-established methods, including PANTHER, SNPs&GO, PolyPhen-2, SIFT, PROVEAN, FATHMM, and MutPred2, with only PMut, VariPred and PON-P2 showing superior performance. Nonetheless, the difference between XGBMut, PMut, and VariPred was negligible, with less than a two percentage point variation across the evaluated metrics. PON-P2 demonstrated a more pronounced performance advantage, with at least 7% higher accuracy and a 3% improvement in ROC-AUC compared with all other methods, including XGBMut (Figure 2B,C). Notably, the ROC-AUC output of VariPred is not shown in Figure 2B, as it provides a class label rather than a probability score, making the calculation of AUC-ROC unfeasible.

PON-P2’s advantage over other methods could be attributed to its limited analysis coverage, as it failed to classify 43% of the test set. This observation is consistent with previous findings by Niroula et al., the developers of PON-P2, who reported a prediction coverage of approximately 62%.^49^ Like PON-P2, PANTHER also demonstrated limited coverage within the test set, successfully classifying only 72.6% of the mutations. In contrast, all other methods, including XGBMut, achieved near-maximal coverage.

Additionally, even when considering the robust MCC metric,^22,50^ XGBMut outperforms well-established methods like PANTHER, SNPs&GO, PolyPhen-2, SIFT, PROVEAN, FATHMM, and MutPred2, while closely matching the performance of state-of-the-art methods such as VariPred (Table 1). The MCC value obtained for VariPred in our study, 0.678, closely aligns with the benchmark analysis conducted by its developers, Lin et al., which reported a value of 0.714. The minor difference in MCC could be attributed to variations in the test set used in their analysis, which included only 21,125 mutations from ClinVar.^11^ In comparison, the MCC value for XGBMut in our study was 0.651, thus reaffirming its robustness and competitiveness in the field of functional prediction. Overall, our findings demonstrate the robustness and effectiveness of XGBMut in correctly identifying deleterious mutations. This analysis positions XGBMut as a highly competitive tool for predicting the functional impact of missense mutations. The model proposed in this study thus serves as a valuable tool for screening potentially harmful mutations, facilitating informed decision-making in both genetic research and clinical applications.

Independent evaluations were conducted by López-Ferrando et al., Pejaver et al., and Bendl et al. assessed at least nine functional prediction algorithms using extensive validation sets sourced from databases such as SwissVar, ClinVar, and the Protein Mutant Database.^7,8,15^ These studies reported accuracy rates ranging from 60% to 81% and ROC-AUC values between 55 and 87%. Our findings are consistent with these results, showing accuracy rates between 73 and 90% (Figure 2B) and ROC-AUC values ranging from 81 to 94% (Figure 2B).

### 3.6 Optimization of the Algorithm and Development of the XGBMut Software

The optimization of the proposed algorithm significantly improved its efficiency and usability. After the inclusion of 36 predictor variables that were not incorporated into the final model and refactoring the search function for the automatic extraction of predictor variables, the algorithm achieved a remarkable 230% performance improvement. This enhancement was quantified by measuring the runtime during the evaluation of a test case from the HumsaVar-derived test set.

The final proposed algorithm is designed to accept a tabular text file containing a set of mutations and their respective UniProt ID identifiers. Once the input file is received, the algorithm then identifies and removes all invalid mutations submitted, which can be optionally saved and exported as a *.csv file. Then, the optimized function for extracting predictor variables is called by the algorithm, loading the reduced databases and substitution matrices, from which the function obtains the corresponding variable values for each mutation. Upon completion of this stage, the data frame containing all extracted predictor variables may be saved and exported as a *.csv file. This functionality offers users customizable access for further analysis, including training and validating their own models as desired.

The final proposed model is subsequently loaded to classify mutations based on their respective predictor variables and to compute the probability of a mutation being deleterious. This is achieved using the *predict* and *predict_proba* functions available in the XGBoost library. Ultimately, the algorithm generates a *.csv file containing the probability of each mutation being deleterious and the corresponding predicted class.

**Figure 3:**
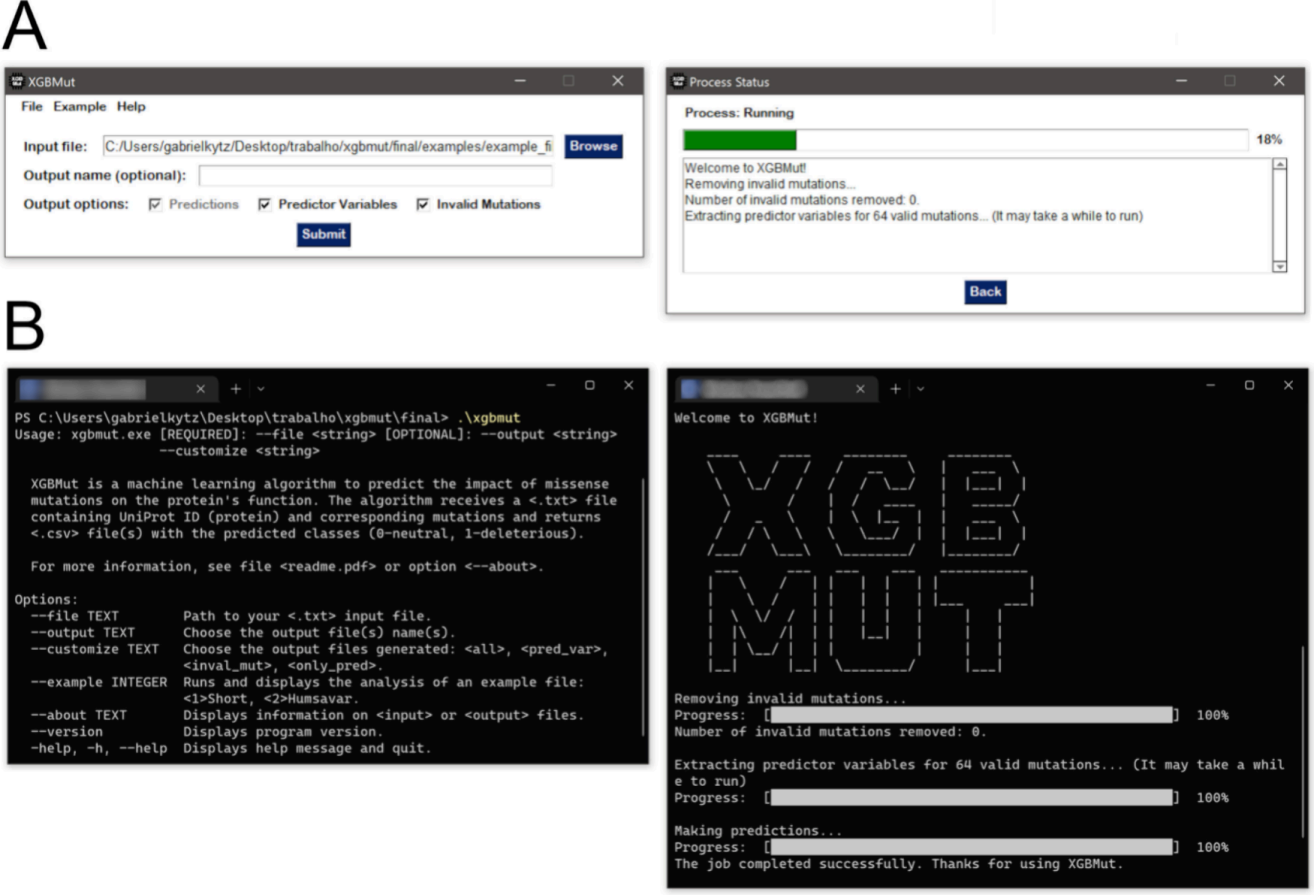
Graphical user interface and command-line interface of the XGBMut software. (A) Graphical user interface. The main menu of the program is on the left, while the analysis loading menu is on the right. (B) Command-line interface. The image corresponds to the Windows version of the interfaces.

To enhance user interaction with the previously described algorithm, two interfaces have been developed that are compatible with both Windows and Linux operating systems. The graphical interface is designed to be intuitive and user-friendly, assisting users, such as doctors and wet-lab researchers, who may not be familiar with command-line tools. As illustrated in Figure 3A, this interface consists of two main sections: an initial menu for file loading and customization, and a loading menu that displays the progress of the analysis. It supports standard analyses and allows for output customization, enabling users to select filenames and components according to their preferences. Additionally, a help menu and example usage options are included to enhance the user experience. Furthermore, a command-line interface is also available for users who are experienced with it, as depicted in Figure 3B.

The maximum RAM usage during testing was kept to a minimum, ensuring compatibility with most computers. Unlike many currently available methods, XGBMut does not require installation, as the executable package includes the Python interpreter and all of the necessary modules into a single executable file. Additionally, the local availability of XGBMut eliminates the need for a web server dependency. As a result, XGBMut presents a novel and integrated approach that combines multiple data sources with optimized runtime and competitive performance, offering a comprehensive framework for predicting the effects of missense mutations. Its user-friendly platform, designed to enhance the user experience, coupled with detailed documentation, makes it an accessible solution for nonspecialists, effectively overcoming the limitations of existing software in the field.

Notably, XGBMut stands apart from most available methods by not only providing the likelihood of deleterious effects for a given set of mutations but also generating a comprehensive output file. This file includes all invalid mutations in the data set, along with the predictor variables used in the model’s predictions, thereby enhancing transparency and the interpretability of results. Finally, the compiled databases and substitution matrices used by XGBMut are fully accessible to users, offering additional resources for further analysis and research.

The XGBMut software will be made available upon publication at the following link: https://github.com/gabrielkytz2/XGBMut/. A complete documentation, including a detailed explanation with step-by-step usage instructions and illustrative screenshots, is available in the GitHub repository to guide users in effectively using the software.

## 4 Conclusions

With over 4 million genetic variants already identified and many more being discovered every year, the need for efficient and scalable methods to analyze these variants is invaluable for diagnosing rare diseases and developing targeted treatments for genetic conditions.^6^ In response to this challenge, we developed a novel functional prediction algorithm named XGBMut, which automatically extracts predictor variables from databases and substitution matrices to efficiently and accurately classify mutations as neutral or deleterious. The algorithm demonstrates competitive performance compared with other widely used methods for the same purpose, underscoring its viability in the field. Furthermore, two user interfaces were developed, including a graphical interface that enables intuitive and user-friendly interaction with the proposed algorithm without the need for installation. This design facilitates accessibility for professionals from various fields, such as doctors and wet-lab researchers. Unlike many currently available methods, XGBMut eliminates the need for an online web server dependency as well as the installation of third-party software and packages. XGBMut is specifically designed to streamline workflows, allowing users—including those without a technical background—to seamlessly integrate genomic analysis into their research. XGBMut is poised to assist in the initial screening of millions of mutations identified in human proteins that remain uncharacterized. Its ability to conduct large-scale and high-performance predictions is strategic for prioritizing missense mutations that are most likely to be pathogenic. By efficiently guiding the design of future experiments, XGBMut not only optimizes time and resource allocation but also enhances the overall productivity of research efforts, ultimately contributing to the discovery of previously uncharacterized pathogenic mutations, the identification of previously unknown disease-causing genes, and improved diagnostic yields for rare genetic disorders.

## Supporting Information Available

(File S1) Detailed description of the data sets used, and features retrieved during the construction of the unified database; (File S2) detailed description of the constructed substitution matrices and the specific features extracted during the creation of the unified database; (Table S1) reduced representation of the unified database; (Table S2) reduced representation of a substitution matrix; (Table S3) reduced representation of training and validation sets; (Table S4) reduced representation of the output file from the automated predictor variable extraction function in the test set; (Table S5) performance of top 10 machine learning models generated by Pycaret; (Table S6) performance of artificial neural networks models; (Table S7) performance of the full model and its simplified forms (PDF)

## Notes

The authors are committed to providing regular updates to the software to ensure its compatibility with current operating systems and relevant packages. Updates will include bug fixes and performance improvements. Whenever necessary, deprecated dependencies will be replaced, and the software will be tested to maintain optimal functionality. Major updates will be detailed in the documentation.
