# Supplementary material for: XGBMUT: Predicting the Functional Impact of Missense Mutations Using an Extreme Gradient Boost Classifier
Source: ACS Omega. 2025 Feb 19;10(8):8349–60. doi: 10.1021/acsomega.4c10179 (PMC11886911; doi:10.1021/acsomega.4c10179)
Supplement: Supplementary file 1 — ao4c10179_si_001.pdf [file ao4c10179_si_001.pdf]

XGBMUT: PREDICTING THE FUNCTIONAL IMPACT OF MISSENSE MUTATIONS USING AN EXTREME GRADIENT BOOST CLASSIFIER

Gabriel Rodrigues Coutinho Pereira[1,2&,*] Loiane Mendonça Abrantes Da Conceição[3,&], Bárbara de Azevedo Abrahim-Vieira[1], Carlos Rangel Rodrigues[1], Lucio Mendes Cabral[2], Ricardo Limongi França Coelho[4], Joelma Freire De Mesquita[3].

[1]Laboratory of Molecular Modeling and QSAR, Faculty of Pharmacy, Federal University of Rio de Janeiro. 373 Carlos Chagas Filho Avenue. 21941-170. Rio de Janeiro, Rio de Janeiro, Brazil.

[2]Laboratory of Industrial Pharmaceutical Technology, Faculty of Pharmacy, Federal University of Rio de Janeiro. 373 Carlos Chagas Filho Avenue. 21941-170. Rio de Janeiro, Rio de Janeiro, Brazil.

[3]Laboratory of Bioinformatics and Computational Biology, Biomedical Institute, Federal University of the State of Rio de Janeiro. 296 Pasteur Avenue. 22290-240. Rio de Janeiro, Rio de Janeiro, Brazil.

[4]Faculty of Business Administration, Accountability and Economics, Federal University of Goiás.No number, Samambaia Street. 74001-970. Goiânia, Goiás, Brazil.

[&]These authors contributed equally to this work.

*Corresponding author: gabrielrodriguescp@gmail.com

1. SUPPORTING INFORMATION

S1 File. Detailed description of the datasets used, and features retrieved during the construction of the unified database.

The following paragraphs outline the molecular features used in developing the predictive model, along with their corresponding representations in the unified database, which are provided in parentheses.

DescribeProt is a database containing structural and functional descriptors at the amino acid level for approximately 20,000 human proteins. Included descriptors encompass information on evolutionary conservation (*MMseq2_conservation_level* and *MMseq2_conservation_score*), secondary structure (*PSIPRED_helix* and *PSIPRED_strand*), solvent accessibility (*ASAquick_normscore*), cell recognition motifs (*MoRFchibiScore*), intrinsically disordered regions (*DFLpredScore* and *VSL2_score*), as well as regions interacting with other proteins (*SCRIBERscore* and *DisoPROscore*), DNA (*DisoDNAscore* and *DRNApredDNAscore*), and RNA (*DRNApredRNAscore* and *DisoRNAscore*) [1].

UniProt is a unified database of protein information compiled from various biological databases, containing data on over 20,000 human proteins. Lists for each protein were derived from UniProt, containing nucleotide binding sites (*Nucleotide binding* and *DNA binding*), calcium binding sites (*Calcium binding*), metal binding sites (*Metal binding*), cofactor binding sites (*Binding site*), signal peptide residues (*Signal peptide*), disulfide bond-forming residues (*Disulfide bond*), and glycosylation sites (*Glycosilation*). The number of interactions each protein engages in was also included as a predictive variable (*Interacts with*) [2].

Gene Ontology (GO) is a database containing gene and biological product information, with functional annotations allowing the classification of human protein function, cellular localization, and involvement in biological processes. Counts of the following GO descriptor classes were included for each protein: involvement in biological processes (*go_involved*), enabling biological processes (*go_enable*), activating biological processes (*go_active*), and presence in cellular compartments and tissues (*go_located*) [3].

PhosphoSitePlus is a curated database providing biological information on amino acid-level post-translational modifications, constructed through text mining of scientific articles from PubMed. Information obtained from PhosphoSitePlus includes glycosylation sites (*O-GalNAc_site*, *O-GlcNAc_site*), ubiquitination sites (*Ubiquitination_site*), phosphorylation sites (*Phosphorylation_site*), sumoylation sites (*Sumoylation_site*), acetylation sites (*Acetylation_site*), methylation sites (*Methylation_site*), as well as protein regions associated with diseases (*Disease-associated_sites*) and regulatory sites (*Regulatory_sites*) [4].

Catalytic Site Atlas (CSA) is a database containing information on catalytic residues in proteins [5]. Catalytic amino acids for each protein obtained from CSA were included in the *catalytic_site* variable. Finally, the Conserved Domain Database (CDD) provides information on evolutionarily conserved domains in proteins [6]. Amino acids comprising conserved domains obtained from CDD were included in the *conserved_domain* variable.

S2 File. Detailed description of the constructed substitution matrices and the specific features extracted during the creation of the unified database.

Initially, we compiled BLOSUM62, which is a well-established matrix built from the probability of amino acid substitution occurrence in alignments of multiple homologous sequences [7].

Then, 7 binary matrices were constructed to characterize changes in physicochemical properties between the original and mutated amino acids. From these properties, different individual matrices were created containing information on changes in *aromaticity, polarity, charge, acidity, hydrophobicity*, and *size*, as well as substitutions to/from proline and cysteine [8]. Substitutions that resulted in a change to a given property were assigned a value of one in the matrix, whereas substitutions that did not alter the property received a value of zero.

An additional matrix was also created containing the frequency of amino acid substitutions in mutations known to be deleterious or neutral in the HuVarBase database, which contains approximately 720,000 classified mutations [9].

Then, we compiled the ProTherm database, which stores experimentally derived thermodynamic information for over 7.000 mutations. The average values of free energy changes

($\Delta\Delta G\_H2O$, $\Delta\Delta G$, ΔTm) for each amino acid substitution, obtained from the ProTherm database, were also used to construct 3 substitution matrices. Additionally, binary matrices were constructed for stabilizing or destabilizing mutations [10]. Substitutions that led to a change in the free energy measurement were assigned a value of one in the matrix, while those that did not affect the property were assigned a value of zero.

The Position Specific Scoring Matrices (PSSM) were obtained from the DescribeProt database. These matrices contain occurrence probabilities for amino acid substitutions. The PSSM matrix is constructed from alignments of multiple homologous sequences, like the BLOSUM-62 matrix but weighted by the affected position in the amino acid sequence [1].

Finally, four individualized numeric matrices were constructed containing differences in values of *molecular mass, number of atoms, volume,* and *hydrophobicity* for each possible amino acid substitution [11].

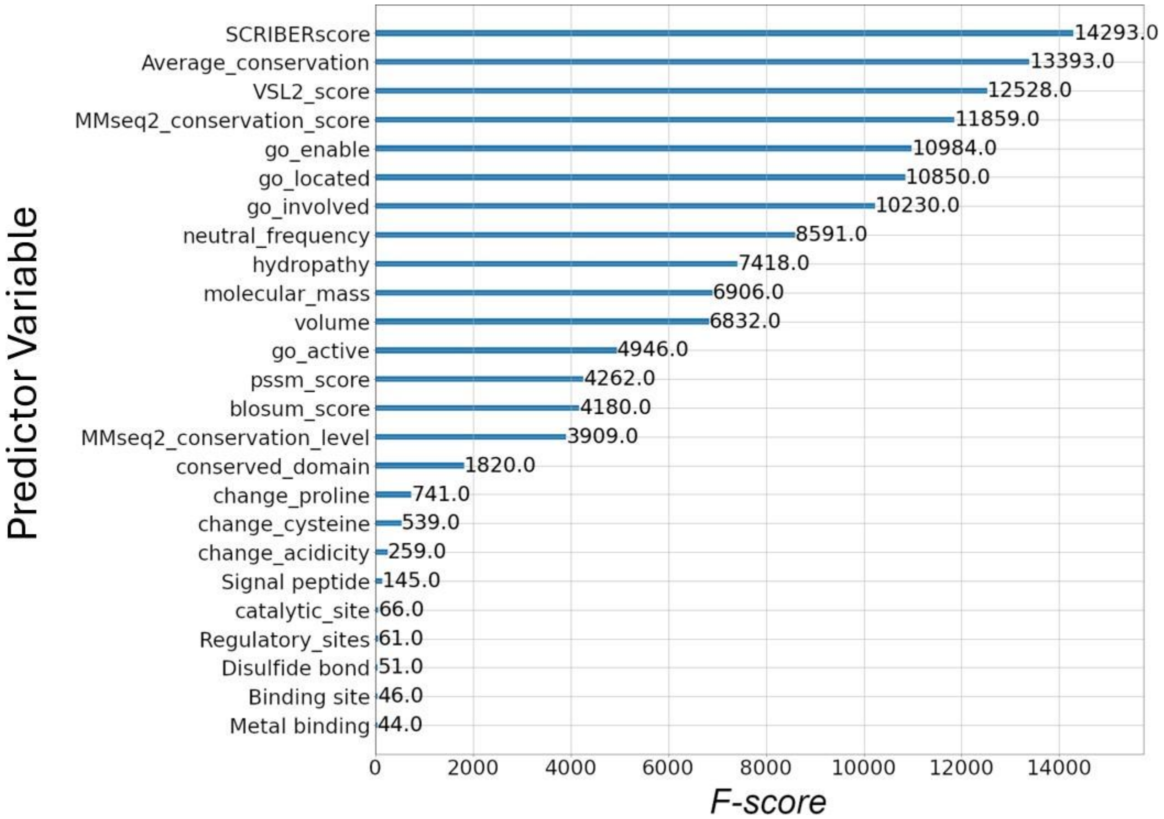

S1 Figure. Importance of Predictor Variables for the Final Model. The importance of each predictor variable was calculated using the *F-score* metric, which represents the number of times a particular feature is used to split data across all trees in the model, reflecting its overall contribution to the model's predictions. Higher F-scores indicate greater importance.

S1 Table. Reduced representation of the unified database.

Index	ACC_entry	ASAquick_normscore	...	Interacts with	seq
A0A024R1R8	A0A024R1R8	[0.75. 0.58. 0.61...]	…	0	MSSHEGG...
A0A024RBG1	NUD4B	[0.69. 0.39. 0.52...]	…	0	MMKFKPN...
A0A075B6H5	A0A075B6H5	[0.69. 0.50. 0.36...]	…	0	METVVTTL...
A0A075B6H7	A0A075B6H7	[0.72. 0.57. 0.35...]	…	0	MEAPAQLL...
A0A075B6H8	A0A075B6H8	[0.65. 0.51. 0.36...]	…	0	MDMRVPA...
…	…	…	…	…	…

[...]: Table continuation not shown. *ACC_entry:* refers to protein entries in the UniProt database.

S2 Table. Reduced Representation of a Substitution Matrix

Index	A	C	D	E	F	...	R	S	T	V	W	Y
**A**	0.0	2.5	2.3	2.9	8.5	…	4.0	1.4	1.1	3.5	0.0	0.6
**C**	3.0	0.0	19.8	0.0	0.0	…	2.6	2.9	3.2	0.8	0.0	0.0
**D**	3.2	12.7	0.0	3.0	5.7	…	3.6	3.8	2.7	2.6	7.6	8.4
**E**	1.2	4.7	1.4	0.0	2.7	…	12.0	1.9	5.0	7.8	5.6	3.3
**F**	4.6	72.0	0.0	14.6	0.0	…	0.0	0.8	0.0	13.0	2.9	4.4
**…**	…	…	…	…	…	…	…	…	…	…	…	…

[...] Table continuation not shown. Amino acids are represented by their one-letter code in both row and column headers. The matrix captures all 20x20 possible amino acid substitutions.

S3 Table. Reduced Representation of Training and Validation Sets

ACC	*AA_change*	*Class*
Q96NU1	R207Q	0
Q96NU1	R28Q	0
Q96NU1	R220Q	0
Q96NU1	R41Q	0
Q96NU1	D234N	0
...	...	...

[...] Table continuation not shown. *ACC:* refers to protein entries in the UniProt database. *AA_change*: denotes the amino acid substitution associated with the mutation.

S4 Table. Reduced Representation of the Output File from the Automated Predictor Variable Extraction Function in the Test Set.

ACC	AA_change	ASAquick_normscore	...	Volume	Hydropathy	Class
P04217	H52R	0.24	…	20.2	-1.3	0

P04217	H395R	0.21	…	20.2	-1.3	0
Q9NQ94	V555M	0.10	…	22.9	-2.3	0
Q9NQ94	A558S	0.29	…	0.4	-2.6	0
P01023	R704H	0.19	…	-20.2	1.3	0
...	...	...	…	...	...	...

[...] Table continuation not shown. *ACC:* refers to protein entries in the UniProt database. *AA_change*: denotes the amino acid substitution associated with the mutation.

S5 Table. Performance of top 10 Machine Learning Models Generated by Pycaret.

Min-Max scaling	Algorithm	Accuracy	AUC-ROC	Recall	Precision	F1-score
no	xgboost	0.819	0.890	0.813	0.782	0.797
no	lightgbm	0.814	0.885	0.815	0.773	0.793
yes	xgboost	0.809	0.880	0.781	0.783	0.782
yes	lightgbm	0.809	0.878	0.790	0.777	0.784
yes	gbc	0.800	0.871	0.807	0.753	0.779
yes	gbc	0.799	0.870	0.805	0.753	0.779
yes	et	0.797	0.857	0.737	0.786	0.761
yes	et	0.787	0.849	0.724	0.775	0.749
no	ada	0.782	0.854	0.797	0.730	0.762
no	ada	0.782	0.854	0.802	0.727	0.763

xgboost: Extreme gradient boosting; *lightgbm:* light gradient boosting; *gbc*: gradient boosting classifier; *et*: extra trees; *ada*: adaptive boosting.

S6 Table. Performance of Artificial Neural Networks Models.

Architecture[1]	Accuracy	AUC-ROC	Recall	Precision	***F1-score***
(6, 6)	0.758	0.836	0.758	0.763	0.759
(12, 8)	0.758	0.831	0.758	0.763	0.758
(8, 8, 8)	0.759	0.836	0.758	0.763	0.759
(8, 6, 4)	0.758	0.838	0.758	0.763	0.759
(12, 8, 6, 4)	0.758	0.842	0.758	0.763	0.759

[1] Refers to the structure of the neural network, represented by the number of neurons in each hidden layer.

S7 Table. Performance of the full model and its simplified forms.

Predictor Variables (N°)	Accuracy	AUC-ROC	Recall	Precision	***F1-score***
61[1]	0.828	0.897	0.828	0.827	0.827
55	0.826	0.896	0.826	0.826	0.826

50	0.827	0.896	0.827	0.827	0.827
45	0.825	0.895	0.825	0.825	0.825
40	0.827	0.897	0.827	0.827	0.827
35	0.827	0.896	0.827	0.827	0.827
30	0.828	0.897	0.828	0.827	0.827
25	0.828	0.898	0.828	0.828	0.828
20	0.827	0.897	0.827	0.827	0.827
15	0.824	0.894	0.824	0.824	0.824
10	0.784	0.852	0.784	0.784	0.784
5	0.752	0.827	0.752	0.756	0.763

[1] Complete model prior to the application of Recursive Feature Elimination (RFE).

2. REFERENCES

1. Zhao B, Katuwawala A, Oldfield CJ, et al. DescribePROT: Database of amino acid-level protein structure and function predictions. *Nucleic Acids Research*. 2021;49(D1):D298-D308. doi:10.1093/nar/gkaa931

2. UniProt Consortium. UniProt: the universal protein knowledgebase in 2021. *Nucleic Acids Research*. 2021;49(D1):D480-D489. doi:10.1093/nar/gkaa1100

3. Carbon S, Douglass E, Dunn N, et al. The Gene Ontology Resource: 20 years and still GOing strong. *Nucleic Acids Research*. 2019;47(D1):D330-D338. doi:10.1093/nar/gky1055

4. Hornbeck PV, Zhang B, Murray B, Kornhauser JM, Latham V, Skrzypek E. PhosphoSitePlus, 2014: Mutations, PTMs and recalibrations. *Nucleic Acids Research*. 2015;43(D1):D512-D520. doi:10.1093/nar/gku1267

5. Ribeiro AJM, Holliday GL, Furnham N, Tyzack JD, Ferris K, Thornton JM. Mechanism and Catalytic Site Atlas (M-CSA): A database of enzyme reaction mechanisms and active sites. *Nucleic Acids Research*. 2018;46(D1):D618-D623. doi:10.1093/nar/gkx1012

6. Chen JW, Romero P, Uversky VN, Keith A. Conservation of Intrinsic Disorder in Protein Domains and Families: I. A Database of Conserved Predicted Disordered Regions. *J Proteome Res*. 2008;5(4):879-887. doi:10.1021/pr060048x.Conservation

7. Eddy SR. Where did the BLOSUM62 alignment score matrix come from? *Nature Biotechnology*. 2004;22(8):1035-1036. doi:10.1038/nbt0804-1035

8. Boyle J. *Lehninger Principles of Biochemistry*. Vol 1. (Nelson D, Cox M, eds.). W.H. Freeman and Company; 2005.

9. Ganesan K, Kulandaisamy A, Binny Priya S, Michael Gromiha M. HuVarbase: A human variant database with comprehensive information at gene and protein levels. *PLoS ONE*. 2019;14(1):1-7. doi:10.1371/journal.pone.0210475

10. Nikam R, Kulandaisamy A, Harini K, Sharma D, Michael Gromiha M. ProThermDB: Thermodynamic database for proteins and mutants revisited after 15 years. *Nucleic Acids Research*. 2021;49(D1):D420-D424. doi:10.1093/nar/gkaa1035

11. Wang X, Weber GF. Quantitative Analysis of Protein Evolution: The Phylogeny of Osteopontin. *Frontiers in Genetics*. 2021;12(August):1-9. doi:10.3389/fgene.2021.700789
